# Needle Arthroscopy in Orthopedic Practice: Clinical Applications, Evidence Architecture, and Translational Gaps—A Scoping Review

**DOI:** 10.3390/jcm15197608

**Published:** 2026-09-30

**Authors:** Hubert Makowski, Piotr Łukowiak, Karolina Michno, Maciej Ciesielski, Jan Mianowski, Michał Maciejowski, Błażej Wójtowicz, Riccardo Compagnoni, Pietro Simone Randelli, Marcin Domżalski, Jędrzej Lesman

**Affiliations:** 1 Department of Orthopaedics and Traumatology, Medical University of Łódź, 90-419 Łódź, Poland; hubert.makowski@stud.umed.lodz.pl (H.M.); piotr.lukowiak@stud.umed.lodz.pl (P.Ł.);; 2 Department of Orthopaedics and Traumatology, University of Milan, 20122 Milan, Italy; 3 IRCCS Istituto Ortopedico Galeazzi, 20157 Milan, Italy

**Keywords:** needle arthroscopy, in-office arthroscopy, diagnostic arthroscopy, evidence architecture, implementation, minimally invasive surgery, PRISMA-ScR

## Abstract

**Background:** Needle arthroscopy can alter not only portal size but also the diagnostic and treatment pathway by enabling direct visualization under local anesthesia and selected limited interventions. Its diffusion across joints, however, may be outpacing clinical validation. **Methods:** This PRISMA-ScR-guided review mapped PubMed/MEDLINE-indexed primary publications from database inception through 31 July 2026. The quantitative flow is therefore an auditable but incomplete evidence map rather than a complete census of the technology; studies indexed exclusively outside PubMed may have been missed. Backward reference-list screening was used as a completeness check, while earlier Scopus, Web of Science, and Cochrane histories were retained as sensitivity sources and not pooled into the quantitative flow. Two reviewers independently screened and charted records using a shared form. Reports were de-duplicated by PMID, DOI, and normalized title and characterized by anatomy, design, application, platform, outcomes, harms reporting, study-design hierarchy, and principal methodological limitation. Technical or cadaveric feasibility was analyzed separately from clinical and comparative evidence. **Results:** The final map comprised 105 unique primary publications: 49 technical, 41 clinical, and 15 cadaveric. Technical and cadaveric reports (64/105, 61.0%) outnumbered clinical reports (41/105, 39.0%), limiting estimation of clinical effectiveness. Ninety publications (85.7%) appeared from 2021 onward; the 2026 count is incomplete because the search ended on 31 July. For descriptive display across the complete map, nine reports (8.6%) were in Levels I–II and 93 (88.6%) in IV–V; among the 41 clinical reports, 29 (70.7%) were IV–V and 12 (29.3%) were I–III. NanoScope accounted for 87 of 107 platform mentions (81.3%). Diagnostic and second-look applications had the most consistent comparative rationale, but lesion-specific estimates varied by joint and reference standard. Reported harms were generally minor where surveillance was described, but rare-harm inference was limited by small samples and inconsistent adverse-event ascertainment. **Conclusions:** The field remains promising but unproven: technical breadth exceeds comparative clinical validation. Because the quantitative evidence map is PubMed/MEDLINE-only and dominated by technical, cadaveric, and IV–V reports, current evidence is insufficient to estimate clinical effectiveness or justify replacement of MRI or conventional arthroscopy. Needle arthroscopy should therefore be viewed as an indication-specific complement and a hypothesis-generating technology rather than a replacement platform. Multicentre comparative studies and randomized trials, where ethically and practically feasible, are needed to test patient benefit and clinical application.

## 1. Introduction

Needle arthroscopy (NA) is more than a smaller arthroscope. By combining needle-sized access, portable imaging, and miniaturized instruments, it can move direct intra-articular visualization from the operating room to an office, procedure room, or wide-awake surgical pathway. The literature uses overlapping labels such as nano-arthroscopy, nanoscopy, in-office needle arthroscopy, and office-based arthroscopy. In this review, needle arthroscopy is the umbrella term; alternative labels are retained only when they identify the wording of an individual report [1,2,3,4,5,6,7].

This shift creates three distinct value propositions that should not be conflated. First, NA may provide problem-solving diagnosis when symptoms, examination, and magnetic resonance imaging (MRI) disagree. Second, it may reduce access morbidity and anesthetic burden for selected low-complexity procedures. Third, it may reorganize the care pathway by shortening time to a definitive decision, reducing operating-room dependence, or avoiding downstream imaging. A device can be feasible without being diagnostically valid, diagnostically valid without improving patient outcomes, and clinically effective without being cost-effective in a different health system.

The literature has expanded from diagnostic knee and shoulder visualization to meniscal surgery, cartilage assessment, biceps procedures, labral repair, ankle impingement, ligament stabilization, second-look assessment, septic-joint management, and fracture reduction. Yet the evidentiary requirements change along this translation pathway. Cadaveric visibility and technical completion establish possibility; comparative accuracy establishes diagnostic performance; randomized or well-controlled studies are needed for clinical-effectiveness claims; and implementation studies are needed to determine whether a new pathway is scalable and economically defensible.

Earlier reviews were predominantly diagnostic, platform-specific, or joint-focused [2,3,6,7]. A 2026 meta-analysis of nine comparative studies (503 patients) reported similar procedural completion, diagnostic success, and complication rates relative to conventional arthroscopy, but certainty ranged from moderate to very low and evidence for therapeutic equivalence remained limited [4]. A separate safety review identified only minor reported complications, although the underlying studies were observational and not designed to exclude uncommon harms [5]. The unresolved question is therefore not whether NA can be used, but for which indication, at what point in the pathway, and with what level of evidentiary confidence.

This scoping review addressed four questions: (1) how is the evidence distributed across joints, applications, study designs, and platforms; (2) which use cases have progressed from technical feasibility to comparative clinical validation; (3) what limits inference regarding safety, generalizability, and implementation; and (4) which research priorities are most likely to convert technical innovation into clinically meaningful evidence.

## 2. Materials and Methods

### 2.1. Review Design and Eligibility Framework

**Figure 1:**
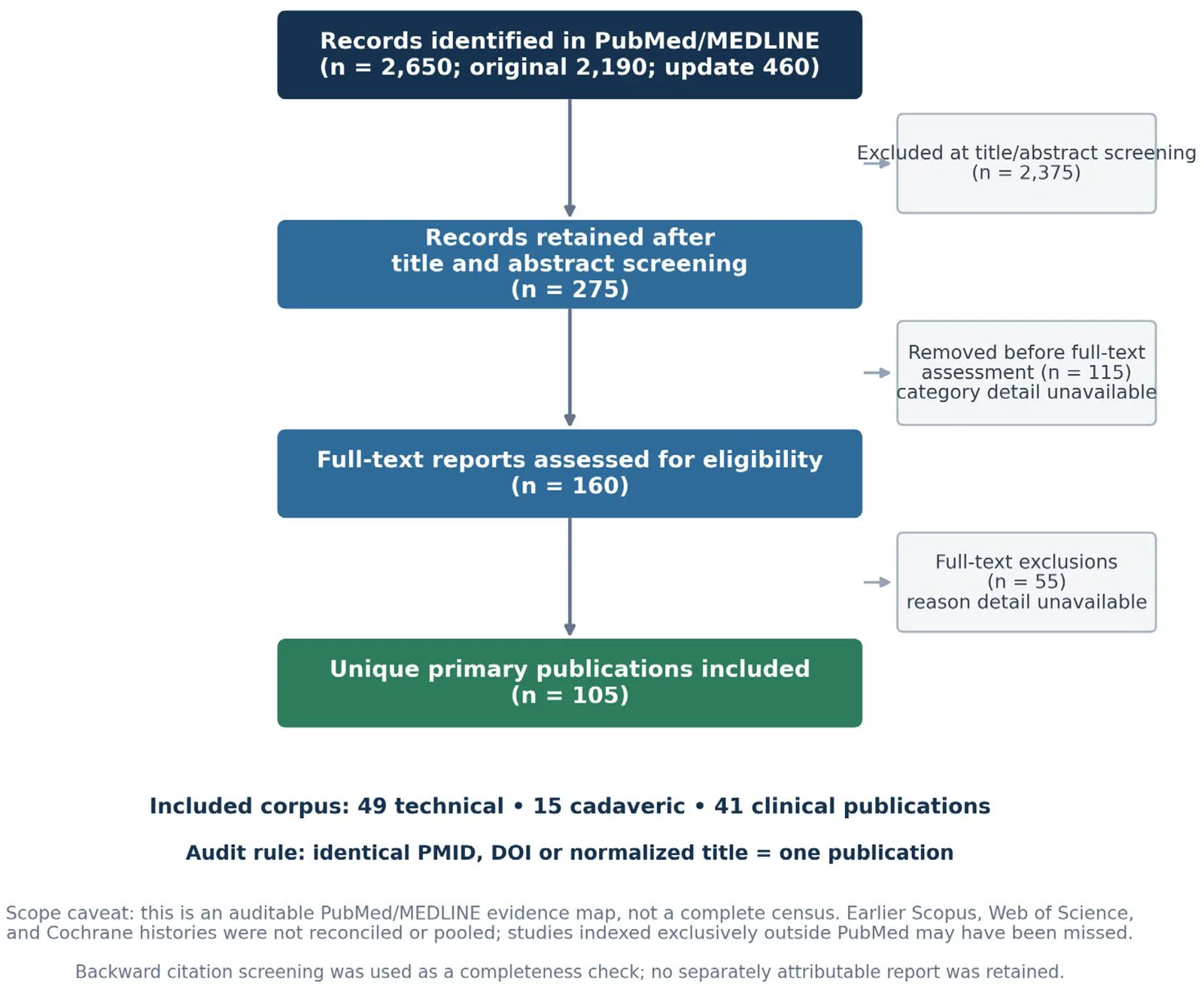
Audited but incomplete PubMed/MEDLINE study-selection flow. Counts were reconstructed from the two-stage PubMed/MEDLINE source workbook and corrected after record-level de-duplication. The included corpus comprised 49 technical, 15 cadaveric, and 41 clinical publications. Earlier Scopus, Web of Science, and Cochrane histories were not reconciled or pooled; studies indexed exclusively outside PubMed may therefore have been missed.

The review was conducted and reported in accordance with the Preferred Reporting Items for Systematic Reviews and Meta-Analyses extension for Scoping Reviews (PRISMA-ScR) [1]. The population–concept–context framework comprised patients or cadaveric specimens representing orthopedic joints (population), needle arthroscopic visualization or intervention (concept), and office, outpatient, operating-room, laboratory, or cadaveric settings (context). Because the purpose was to characterize both clinical use and the translation pipeline, cadaveric and technical reports were eligible but were analyzed separately from patient-level clinical evidence. The protocol was not prospectively registered. The completed PRISMA-ScR checklist is provided as Supplementary Table S2 for publication alongside the manuscript, and the audited study-selection flow is presented in Figure 1.

Eligible primary publications reported an orthopedic diagnostic, therapeutic, technical, feasibility, accuracy, safety, workflow, or economic application of needle arthroscopy. Randomized and non-randomized clinical reports, diagnostic-accuracy studies, case series or reports, technical notes, and cadaveric investigations were included. Veterinary reports, non-orthopedic endoscopy, conference abstracts without a full report, editorials, letters, and review articles were excluded from the primary evidence map. Reviews were retained only to frame prior knowledge and to cross-check reference lists. A “report” denotes one publication; it does not necessarily represent an independent cohort.

### 2.2. Information Source and Search Strategy

The auditable primary search used PubMed/MEDLINE from database inception through 31 July 2026. Five terms were entered as separate PubMed all-fields searches and then merged: NanoScope; nanoscopy; “needle arthroscopy”; microarthroscopy; and “in-office arthroscopy”. No language, species, or date filter was applied. The original search contributed 2190 records, and the update contributed 460 records. The archived workbook does not preserve term-level hit counts or the exact execution date of the original search, so those details cannot be reconstructed retrospectively without inventing data. The complete executable terms, available counts, and audit rules are provided in Supplementary Table S1.

Backward reference-list screening of included reports and relevant reviews was used as a completeness check. Candidate records were assessed against the same criteria and were retained in the final map only after PMID/DOI verification. Earlier search histories in Scopus, Web of Science, and the Cochrane Library used the same conceptual blocks as sensitivity sources; because their July 2026 record-level exports could not be fully reconciled with the PubMed dataset, they were not used to calculate the quantitative PRISMA flow. The quantitative results should therefore be interpreted as an auditable but incomplete PubMed/MEDLINE-indexed evidence map: studies indexed exclusively outside PubMed may have been missed, and not every published analysis of needle arthroscopy is necessarily represented.

### 2.3. Selection, De-Duplication, and Data Extraction

Two reviewers independently screened titles and abstracts and prepared candidate inclusion lists; potentially eligible full texts were then assessed independently, with disagreements resolved by consensus. Data charting was performed independently in duplicate using a shared form and reconciled before synthesis. The historical and updated lists were re-audited at record level. Publications with the same PMID, DOI, or normalized title were treated as one report. This process removed three duplicate entries (PMID 39682041, 38235751, and 39077809), completed one previously uncharacterized retrieved study (PMID 40168804), restored the eligible Deirmengian report (PMID 29494711) identified by Reviewer 3, and verified PubMed identifiers for all 105 included publications. Four included reports were co-authored by one or more review authors (Błażej Wójtowicz, Marcin Domżalski, or Jędrzej Lesman; PMIDs 39165867, 41206227, 41630068, and 42443948). For each of these records, the study-design, evidence-architecture, and translation-stage classifications were assigned by a reviewer who was not a co-author of that report and then independently checked by the other member of the screening pair; the archived log retained the pair-level process but not report-level reviewer initials, so we do not retrospectively attribute individual records to named reviewers. These reports were not used as the sole basis for any conclusion.

Data charting captured year, report design, anatomical region, indication, sample size, platform, principal findings, harms reporting, study-design category, and the main methodological limitation. We distinguished a publication/report from an independent study or cohort; the latter could not be established reliably for every specialist-group series. A pooled patient total was intentionally not calculated because the corpus included technical procedures, cadaveric specimens, potentially overlapping cohorts, and multi-joint populations; summing these denominators would imply a precision and independence that the evidence does not possess.

### 2.4. Study-Design Hierarchy and Structured Methodological Characterization

For descriptive comparison of patient-level clinical reports, study design was mapped to the Oxford Centre for Evidence-Based Medicine Levels of Evidence (March 2009) [6]. Sublevels were collapsed to five categories: I, randomized trial; II, prospective comparative or diagnostic-accuracy report with an appropriate reference standard; III, retrospective comparative or prospective non-randomized comparative report; IV, case series or cohort without a comparator; and V, case report or report without clinical comparative evidence. Legacy “IIb” entries were mapped to Level II. Technical, bench, and cadaveric reports were displayed in the V category for the purpose of the evidence-architecture figure only and were not interpreted as clinical Level V evidence. These categories are a study-design hierarchy, not a risk-of-bias, certainty-of-evidence, or recommendation grade.

We did not perform a formal cross-design risk-of-bias assessment because the archived extraction did not preserve complete item-level judgments for every report. Instead, design-matched prompts from RoB 2, QUADAS-2, and MINORS informed a structured methodological characterization. For each report, reviewers recorded whether a comparator or reference standard was present, whether outcome assessment was blinded or independent, whether follow-up and adverse-event surveillance were defined, whether a denominator was available, and the principal limitation. The full characterization is provided in Supplementary Table S1; “not reported” was kept distinct from “reported absent”.

The translation stage was assigned at the report level using explicit rules: Stage 1, technical or preclinical feasibility without patient outcomes; Stage 2, clinical feasibility demonstrated in patients without a comparative reference; Stage 3, comparative diagnostic or therapeutic validation with a reference standard or comparator; and Stage 4, implementation evidence involving multicentre workflow, learning-curve, resource, or economic outcomes. When a report contained more than one element, the highest stage directly supported by its design and outcomes was recorded. These stages are interpretive descriptors of the literature and should not be read as validated measures of evidence maturity.

### 2.5. Synthesis

The synthesis used three complementary axes: anatomical distribution, study architecture (technical, cadaveric, or clinical; design hierarchy; platform), and clinical function (diagnostic or second-look, therapeutic, or care-pathway implementation). Claims from feasibility reports were deliberately separated from comparative effectiveness. Meta-analysis was not planned because interventions, comparators, outcomes, and follow-up were too heterogeneous and because the review question concerned evidence mapping rather than a single pooled treatment effect.

## 3. Results

### 3.1. Study Selection

The two-stage PubMed/MEDLINE search yielded 2650 records. Initial relevance filtering retained 275 records; 115 duplicate, veterinary, or otherwise ineligible records were removed before full-text assessment. Of 160 full-text reports assessed, 55 were excluded. The final evidence map contained 105 unique primary publications [8,9,10,11,12,13,14,15,16,17,18,19,20,21,22,23,24,25,26,27,28,29,30,31,32,33,34,35,36,37,38,39,40,41,42,43,44,45,46,47,48,49,50,51,52,53,54,55,56,57,58,59,60,61,62,63,64,65,66,67,68,69,70,71,72,73,74,75,76,77,78,79,80,81,82,83,84,85,86,87,88,89,90,91,92,93,94,95,96,97,98,99,100,101,102,103,104,105,106,107,108,109,110,111,112]. The manuscript, figures, and Supplementary Table S1 were reconciled against the same PMID-verified dataset (Figure 1). Because the quantitative flow was limited to auditable PubMed/MEDLINE source files, Figure 1 represents an incomplete evidence map rather than an exhaustive census of the field.

### 3.2. Distribution of the Evidence

**Figure 2:**
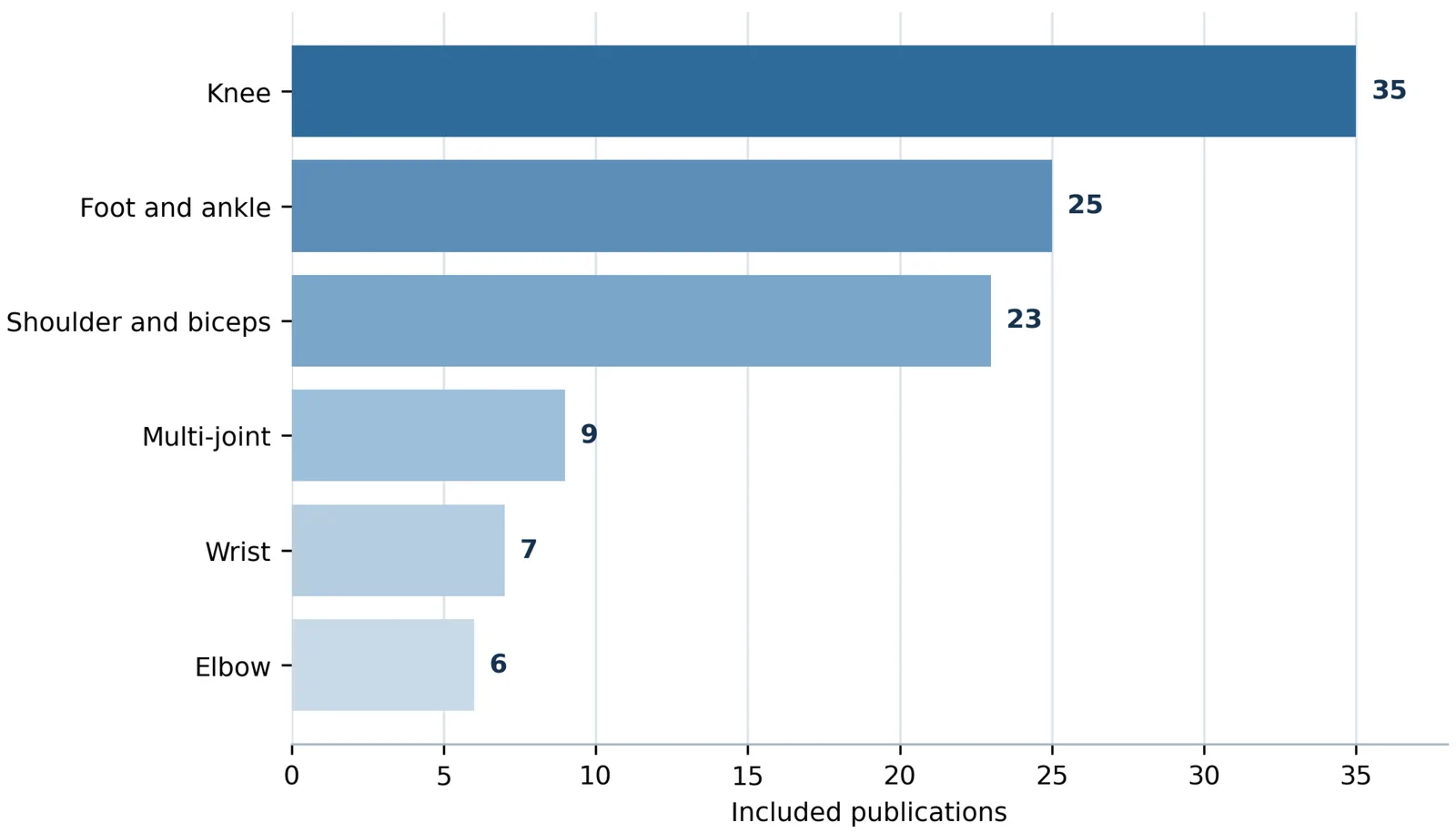
Anatomical distribution of the 105 unique included primary publications.

**Figure 3:**
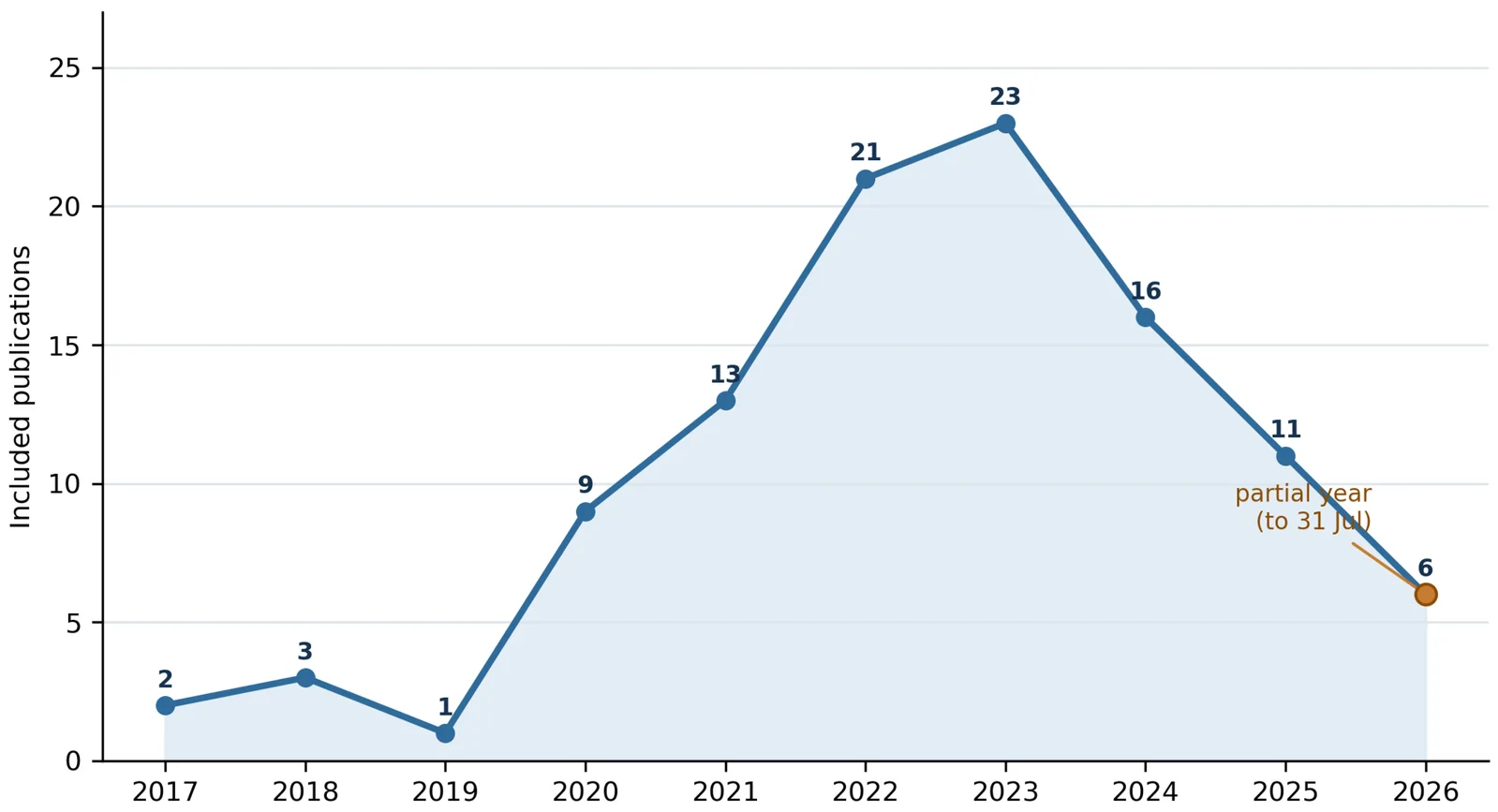
Publication activity by year. The 2026 value is a partial observation period and includes only publications indexed through 31 July 2026; it should not be interpreted as a decline in publication activity.

The knee was the most frequently represented region (*n* = 35), followed by foot and ankle (*n* = 25), shoulder and biceps (*n* = 23), multi-joint applications (*n* = 9), wrist (*n* = 7), and elbow (*n* = 6) (Figure 2). Publication growth was recent and steep: 90 of 105 publications (85.7%) appeared from 2021 onward (Figure 3). The temporal signal therefore reflects rapid diffusion of a platform rather than the accumulation of long-term follow-up.

### 3.3. Evidence Architecture and Platforms

The evidence architecture was weighted toward innovation description. Forty-nine publications were technical, 15 cadaveric, and 41 clinical, meaning that technical and cadaveric reports (64/105, 61.0%) outnumbered patient-level clinical reports. Across the complete map, nine of 105 reports (8.6%) fell in Levels I–II and 93 (88.6%) in the IV–V design categories used for descriptive display. In the clinical subset, 29 of 41 reports (70.7%) were IV–V and 12 (29.3%) were I–III. The single Level I report was a multicentre randomized comparison of partial meniscectomy with needle versus standard arthroscopy [38]. These figures describe study design; they are not a validated certainty grade or a direct measure of clinical benefit. The predominance of technical, cadaveric, and low-level clinical evidence makes a pooled estimate of clinical effectiveness inappropriate.

**Figure 4:**
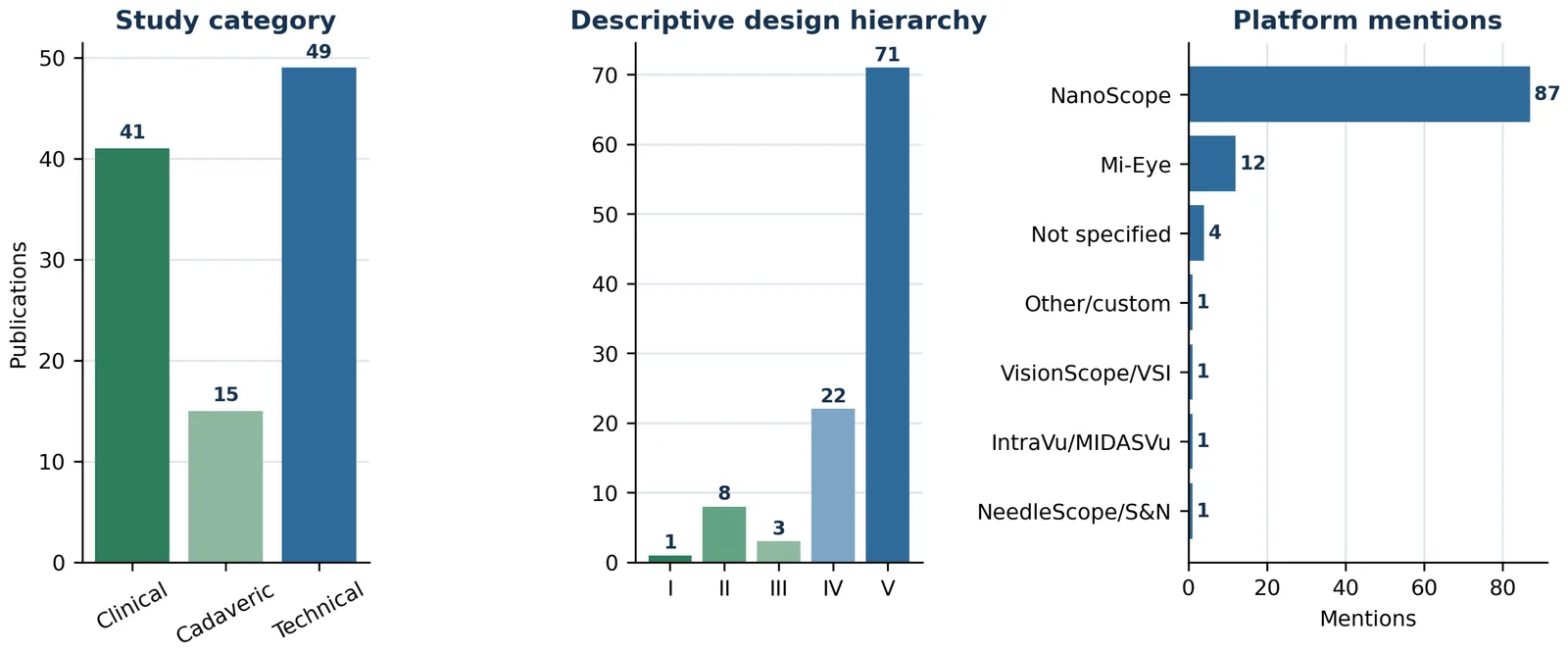
Evidence architecture by study category, descriptive design hierarchy, and reported platform. Platform counts are mentions and are not mutually exclusive; the hierarchy is not a certainty-of-evidence grade.

Platform concentration was also substantial. NanoScope accounted for 87 of 107 platform mentions (81.3%), followed by Mi-Eye (*n* = 12). Four studies did not specify a platform, and one mention each involved VisionScope/VSI, IntraVu/MIDASVu, NeedleScope/Smith & Nephew, and a custom device. Two publications evaluated more than one system. The dominance of a single commercial platform means that observed performance may reflect device-specific optics, instruments, training, and early-adopter expertise rather than a class effect. Conclusions therefore cannot be generalized automatically from the NanoScope literature to all needle-arthroscopy systems (Figure 4).

### 3.4. Joint-Specific Applications

Sensitivity check for author-linked reports. Four included reports were co-authored by review authors. Excluding these four records reduced the foot-and-ankle count from 25 to 23 and the shoulder-and-biceps count from 23 to 21, but did not change the directional conclusions in Table 1: foot and ankle remained characterized by small specialist-centre cohorts and few controls, while shoulder and biceps remained dominated by technical or clinical-feasibility evidence rather than comparative validation. No region changed from a feasibility-dominant to a comparative-validation-dominant architecture. This was a narrative robustness check at the publication level, not a formal leave-one-out analysis of independent cohorts.

### 3.5. Diagnostic Evidence

Diagnostic and second-look use represented the most coherent clinical role. Eight clinical reports used MRI, conventional arthroscopy, or surgical findings as a reference or comparator, but their joints, lesions, timing, blinding, and denominators differed. Across knee, shoulder, and wrist reports, NA often showed high completion and clinically useful concordance for selected structures [11,32,36,94,105,112,113]. The signal was not uniform: visualization depends on portal trajectory, joint volume, a 0° viewing angle, lesion location, and operator experience. “Diagnostic accuracy” should therefore be interpreted as a joint- and lesion-specific property, not as a platform-wide attribute or proof of improved patient outcomes.

The 2026 comparative meta-analysis found no significant difference in diagnostic success or procedure completion between NA and conventional arthroscopy, but estimates were imprecise, and certainty ranged from moderate to very low [4]. The evidence supports NA most strongly when direct visualization is expected to resolve a management-relevant uncertainty—not as routine duplicate testing after an already definitive MRI. Table 2 summarizes the eight reports with a clinical reference standard; no pooled diagnostic estimate was calculated.

### 3.6. Therapeutic and Perioperative Outcomes

Therapeutic scope expanded to partial meniscectomy and repair, cartilage procedures, ankle impingement, ligament stabilization, biceps tenotomy or tenodesis, labral procedures, tendon release, infection management, and fracture reduction. The evidentiary signal was strongest for short-term perioperative burden rather than durable superiority. In the multicentre randomized knee trial, needle-arthroscopic partial meniscectomy reduced opioid consumption in the first 24 h and improved KOOS pain and IKDC scores at 2 and 6 weeks; between-group differences were absent at 12 weeks [38]. Earlier comparative work similarly suggested less early pain, without establishing long-term non-inferiority [35].

Preference and pathway outcomes were promising but context-dependent. Patients often favoured in-office NA when it shortened time to treatment or avoided general anesthesia, while concern centred on limited long-term and revision evidence [40]. Economic studies suggested that savings may arise from avoided MRI, operating-room use, or downstream visits [114,115,116]. However, most cost models were developed in the United States or in single-centre settings. Their estimates are therefore sensitive to local reimbursement, wage structure, MRI availability, equipment utilization, staffing, sterile-workflow requirements, and whether NA replaced rather than duplicated another test; they should not be transported as universal economic conclusions.

Recent publications broadened technical possibility: a custom 2 mm high-definition device underwent optical and cadaveric evaluation [39]; ankle work extended to operative arthroscopy, impingement, and second-look assessment [43,44,61]; shoulder work included labral repair and acromioplasty [79,85]; regional-anesthesia biceps procedures were described in early feasibility or technical studies [75,78,86,88]; and needle-arthroscopy-assisted tibial plateau reduction improved reduction quality in a cadaveric comparison [41]. These studies define a research frontier; they do not, by themselves, establish routine clinical effectiveness.

### 3.7. Safety, Learning Curve, and Industry Involvement

The published safety signal was reassuring but not definitive. A 2025 systematic review of 11 studies (1624 patients) reported joint-specific complication ranges of 0–9.68%; reported events were grade I, and vasovagal reactions were most frequent [5]. Most primary studies, however, were too small and followed patients too briefly to exclude uncommon infection, neurovascular injury, iatrogenic chondral injury, or later reoperation. Absence of observed serious harm in small feasibility cohorts is not equivalent to evidence of safety. A statement that “no complications occurred” was retained as a report-level observation, not converted into a class-wide safety estimate; no pooled complication rate was calculated because adverse-event definitions, denominators, and surveillance periods were inconsistent.

Restricted field of view, unfamiliar 0° optics, lower instrument rigidity, fluid-management constraints, and limited reconstructive capability were recurring barriers. Procedural completion is likely to be operator- and indication-dependent, yet case volume, learning curve, conversion thresholds, and failed attempts were inconsistently reported. In the audited disclosure fields, 51 of 105 reports (48.6%) named a manufacturer or industry relationship, 10 (9.5%) explicitly reported no support or no conflict, and 44 (41.9%) did not report enough information to classify the relationship. This classification was based solely on the conflict-of-interest, funding, acknowledgments, equipment-support, or other relationship statements printed in the report or available in its full text; absence of a statement was coded as not reported or unclear, not as evidence that no relationship existed. These counts are descriptive rather than proof of bias, but concentration in specialist centres, overlap of three review authors with included reports, and frequent manufacturer support create plausible risks of industry-related and publication bias. They limit transportability even when technical success is high.

## 4. Discussion

### 4.1. Principal Findings

The principal finding is an innovation-to-evidence mismatch. The literature has expanded rapidly in volume, anatomy, and procedural ambition, but 93 of 105 reports (88.6%) were in the IV–V design categories used for descriptive display; 64 reports were technical or cadaveric and only 41 were clinical. Only nine reports (8.6%) were in Levels I–II. In the 41-report clinical subset, 29 (70.7%) were IV–V and 12 (29.3%) were I–III. Low-complexity feasibility work is appropriate during early device development, but technical completion or cadaveric visibility cannot establish diagnostic efficacy, therapeutic non-inferiority, or system-level value. The predominance of non-clinical and low-level evidence makes clinical-effectiveness estimation uncertain. Those claims require comparative patient studies with appropriate reference standards, follow-up, and prespecified outcomes.

The translation signal differed by use case more than by device. Problem-solving diagnosis and planned second-look assessment had a clear mechanism of value and the most consistent comparative support, although estimates remained lesion-specific. Selected low-complexity procedures had a plausible early-morbidity advantage, but durable outcomes were sparse. Complex repair and reconstruction remained predominantly at the technical frontier. The 2026 meta-analysis supports comparable diagnostic and procedural performance in selected comparisons, but certainty ranged from moderate to very low and conventional arthroscopy remains the reference for definitive therapeutic intervention [4]. Our “evidence architecture” and translation stages are structured interpretive descriptors, not validated measures of evidence maturity.

### 4.2. Clinical Positioning

The following is an evidence-informed hypothesis for clinical positioning, not a recommendation for routine practice. The relevant question is not whether NA can visualize a joint, but whether direct visualization at that point will change management while exposing the patient to less burden than the next-best alternative. NA may be most plausible when symptoms and imaging disagree, MRI is contraindicated or repeatedly non-diagnostic, postoperative tissue status will alter care, or a limited intervention can be completed with a high probability of success using available instrumentation. These scenarios should be tested prospectively rather than treated as established indications. Nothing in the current evidence justifies replacing MRI or conventional arthroscopy as the default diagnostic or therapeutic platform.

Conversely, conventional arthroscopy remains preferable when treatment requires broad visualization, multiple working portals, traction, high instrument rigidity, complex suture management, extensive debridement, or immediate escalation. Consent should explicitly address incomplete visualization, conversion, and the possibility that an office-based diagnostic procedure may still be followed by conventional surgery. This prevents “minimally invasive” from becoming a surrogate for “clinically sufficient.” Table 3 proofs the positioning in evidence based medicine.

### 4.3. Generalizability and Care-Pathway Value

Generalizability is constrained at both the device and operator levels. NanoScope accounted for 87 of 107 platform mentions (81.3%), while many reports originated from high-volume early-adopter groups. A favourable result for one platform, indication, and expert operator should not automatically be interpreted as a class effect. Device-specific optics, instruments, single-use components, training pathways, and manufacturer involvement may all influence observed performance. Replication across independent centres and platforms should therefore report optic diameter and angle, instruments, fluid strategy, setting, anesthesia, operator case volume, technical completion, conversion, and all commercial relationships.

The economic value proposition also requires a pathway rather than a procedure perspective. NA may create value when it replaces an MRI, operating-room diagnostic arthroscopy, general anesthesia, or additional visits. It may increase cost when it duplicates imaging, is followed by planned conventional arthroscopy, or requires low-utilization capital equipment. Most available models are US-based or single-centre analyses; differences in reimbursement, salaries, MRI access, sterilization standards, and care pathways can reverse the direction of an apparent saving. Future evaluations should state the displaced resource, time horizon, payer perspective, local prices, sensitivity analyses, and downstream consequences rather than report procedure cost in isolation [114,115,116].

### 4.4. Research Priorities and Minimum Reporting Dataset

The next phase should be question-matched rather than technology-led. Diagnostic studies require blinded, lesion-specific reference standards and reporting of non-visualized compartments. Therapeutic studies require procedure-specific non-inferiority margins, conversion criteria, and follow-up long enough to detect reoperation. Where ethically and practically feasible, randomized trials should compare needle arthroscopy with MRI, conventional arthroscopy, or the current standard pathway using patient-relevant outcomes. Multicentre comparative cohorts remain important where randomization is impractical. Implementation studies require multicentre replication, learning-curve analysis, and full pathway costing. A shared minimum dataset would make small studies cumulatively informative and reduce outcome fragmentation (Table 4).

### 4.5. Limitations

This review has several limitations. First, the quantitative PRISMA flow was reconstructed from the auditable PubMed/MEDLINE source files and should be understood as an incomplete evidence map rather than a complete census of the technology. Earlier Scopus, Web of Science, and Cochrane histories were used only as sensitivity sources; their updated record-level exports, term-level hit counts, and original execution date were not preserved, so studies indexed exclusively outside PubMed may have been missed, and the multi-database search cannot be reproduced in full. Second, the protocol was not prospectively registered. Third, deliberate inclusion of technical and cadaveric evidence was necessary to map translation but makes the corpus unsuitable for a single effectiveness estimate. Fourth, the Oxford-derived design hierarchy and translation stages are structured author assessments, not validated certainty or maturity classifications; no cross-design risk-of-bias score was calculated because item-level archival judgments were incomplete. Fifth, the corpus is publication-based: repeated specialist groups and possible cohort overlap could not be resolved for every report, and four reports were co-authored by review authors. Those records were classified by a non-coauthor reviewer and independently checked, but the archived log did not retain report-level reviewer initials. Sixth, adverse-event definitions, denominators, and follow-up were inconsistent, and a report of no observed complications cannot exclude rare harms. Finally, 2026 indexing was incomplete at the 31 July cutoff, and the full-text exclusion archive did not retain mutually exclusive reason counts. A future update should prospectively register its protocol and run synchronized, fully exportable searches across all planned databases from the outset, using a common date window and a reproducible de-duplication log so that cross-database reconciliation is possible. These constraints define the product as an incomplete evidence map and hypothesis-generating readiness assessment, not proof of clinical effectiveness or a basis for replacing MRI or conventional arthroscopy.

## 5. Conclusions

Needle arthroscopy is a promising but unproven technology within a rapidly expanding and uneven evidence base. Among 105 PMID-verified primary publications, 64 were technical or cadaveric, only 41 were clinical, 93 (88.6%) were in the IV–V design categories used for descriptive mapping, and only nine (8.6%) were in Levels I–II. The literature therefore supports technical feasibility and selected, indication-specific diagnostic concordance more strongly than it supports comparative clinical effectiveness, long-term safety, therapeutic equivalence, or economic value. Because the quantitative flow was based on auditable PubMed/MEDLINE files and studies indexed exclusively elsewhere may have been missed, the map is not a complete census of the field. Current evidence does not justify replacing MRI or conventional arthroscopy. NanoScope represented 81.3% of platform mentions, so findings should not be generalized automatically to needle arthroscopy as a class. Progress now depends on independent multicentre comparisons, randomized trials where feasible, lesion-specific reference standards, conversion and learning-curve reporting, standardized harms surveillance, and health-system-specific pathway economics.

## Figures and Tables

**Table 1:** Joint-specific evidence architecture. The comparative evidence signal summarizes the strongest design present; it is not a formal certainty grade or recommendation.

Region	*n*	Dominant Use Cases	Comparative Evidence Signal	Translation-Limiting Gap
Knee	35	Diagnosis; meniscal and cartilage procedures; ligament or second-look assessment; fracture reduction	Prospective diagnostic comparisons and one multicentre randomized therapeutic trial	Durability, conversion, multicentre reproducibility, and procedure-specific non-inferiority
Foot and ankle	25	Osteochondral lesions; impingement; instability; tendoscopy; infection; second-look	Prospective feasibility and early patient-reported outcomes in selected indications	Small specialist-centre cohorts, few controls, and uncertain external validity
Shoulder and biceps	23	Diagnosis; biceps procedures; cuff or labral techniques; acromioplasty; infection	Clinical feasibility for selected wide-awake procedures	Technical literature exceeds comparative outcome evidence; incomplete visualization and learning curve
Wrist	7	Carpal diagnosis; wide-awake arthroscopy; portal-safety studies	Small comparative visualization studies	Limited samples, operator dependence, and sparse patient-centred follow-up
Elbow	6	Diagnosis; tendon release; fracture assistance	Anatomical and procedural feasibility	Evidence remains predominantly cadaveric or technical
Multi-joint	9	Diagnostic pathways; safety; network or workflow outcomes; fracture assistance	Early implementation and broad feasibility signals	Heterogeneous populations, settings, and outcome definitions

**Table 2:** Clinical diagnostic reports with an operative or conventional arthroscopy reference standard. Values are report-level findings; heterogeneity precluded pooling.

Report	Joint/*n*	Reference or Comparator	Main Report-Level Signal	Key Limitation
Deirmengian 2018 [112]	Knee/106	Surgical arthroscopy; MRI comparator	Per-patient agreement 91.5% NA vs. 61.3% MRI; meniscal sensitivity 92.6% vs. 77.8%, specificity 100% vs. 41.7%	Prospective multicentre pilot; operating-room pathway; manufacturer relationships reported
Gill 2018 [11]	Knee/110	Surgical diagnostic arthroscopy	Blinded expert review; kappa 0.766–0.902 for NA vs. surgical arthroscopy, higher than MRI comparisons	Short-term diagnostic study; no patient-outcome follow-up
Wagner 2021 [73]	Shoulder/50	Surgical arthroscopy	NA showed lesion-specific accuracy broadly comparable with MRI for selected structures	Small anesthetized cohort; sensitivity varied by tissue
DeClouette 2022 [105]	Shoulder/knee/35 joints	Surgical arthroscopy	High sensitivity/specificity reported in a retrospective cohort	Single surgeon and centre; blinding and selection incompletely reported
Chowdhury 2023 [77]	Shoulder/22 (20 MRI)	Conventional arthroscopy; MRI comparator	NA sensitivity/specificity: rotator cuff 0.75/1.00; LHB 0.67/0.95; fewer structures visualized than conventional scope	Very small cohort; sequential same-session design; limited generalizability
Nakasa 2023 [32]	Knee meniscus/40	MRI and diagnostic arthroscopy	Three blinded surgeons; moderate inter-reader agreement (kappa 0.517); lesion-level MRI patterns variable	Moderate agreement; reporting of timing and full reference details limited
Moses 2024 [94]	Wrist/20	2.7 mm conventional arthroscopy	No meaningful difference in diagnosis or visualization in sequential surgeon assessment	Small survey-based comparison; no full blinding or patient outcomes
Cabatu 2026 [87]	Shoulder/30	Standard 4 mm arthroscopy; MRI	High agreement for selected biceps and cuff structures; kappa for LHB about 0.73	Single centre; anesthetized pathway; Arthrex support/consultancy reported

**Table 3:** Evidence-calibrated clinical positioning of needle arthroscopy.

Clinical Decision	Evidence-Calibrated Present Role	Claim Not Yet Justified
Equivocal intra-articular diagnosis	Problem-solving direct visualization when the result will change management	Routine replacement of MRI across lesions, joints, and operators
Postoperative or second-look assessment	Low-burden assessment with a predefined management consequence	Surveillance without a decision threshold
Limited meniscal or soft-tissue procedure	Potential reduction in early pain, opioid use, or anesthetic burden in experienced hands	Universal long-term non-inferiority
Complex repair or reconstruction	Adjunctive visualization in selected steps	Replacement of conventional arthroscopy as the default platform
Office-based care pathway	Potential time and resource benefit in a validated sterile workflow	Automatic cost-effectiveness or scalability across health systems

**Table 4:** Proposed minimum reporting dataset for clinical and implementation studies of needle arthroscopy.

Domain	Minimum Items to Report	Why it Matters
Selection	Joint, lesion, symptom duration, prior imaging, and explicit reason for NA	Defines the intended-use population and avoids indication drift
Procedure	Setting, anesthesia, platform, optic, instruments, operator experience, completion, and conversion	Separates device performance from operator and workflow effects
Effectiveness	Reference standard; validated PROMs; pain or opioid use; return to activity; reoperation	Links technical success to patient-relevant benefit
Safety	Prospective denominator, predefined adverse events, infection, neurovascular or chondral injury, 30- and 90-day surveillance	Permits rare-harm aggregation and meaningful comparison
Implementation	Procedure and pathway time, staffing, equipment, sterilization, waste, downstream imaging, and total cost	Tests scalability and whether another resource was genuinely displaced

## Data Availability

The extracted study-level data are provided in Supplementary Table S1.

## Supplementary Materials

The following supporting information can be downloaded at: https://www.mdpi.com/article/10.3390/jcm15197608/s1. Supplementary Table S1—Evidence base audit. Supplementary Table S2—PRISMA-ScR Compliance Checklist.

## Abbreviations

The following abbreviations have been used in this manuscript:

CA	conventional arthroscopy
IONA	in-office needle arthroscopy
MINORS	Methodological Index for Non-Randomized Studies
MRI	magnetic resonance imaging
NA	needle arthroscopy
PRISMA-ScR	Preferred Reporting Items for Systematic Reviews and Meta-Analyses extension for Scoping Reviews
QUADAS-2	Quality Assessment of Diagnostic Accuracy Studies 2
RCT	randomized controlled trial
RoB 2	Risk of Bias 2
