# Supplementary material for: Needle Arthroscopy in Orthopedic Practice: Clinical Applications, Evidence Architecture, and Translational Gaps—A Scoping Review
Source: J Clin Med. 2026 Sep 30;15(19):7608. doi: 10.3390/jcm15197608 (PMC13666524; doi:10.3390/jcm15197608)
Supplement: Supplementary file 1 [file jcm-15-07608-s001.zip › Supplementary_Table_S2.pdf]

PRISMA-ScR Compliance Checklist

Manuscript: “Needle and Nano-Arthroscopy Across Orthopaedic Practice: Clinical Utility, Evidence Maturity, and Translational Gaps—A Scoping Review”

Checklist basis: PRISMA Extension for Scoping Reviews (Tricco et al., 2018). Item descriptions below are concise author-prepared labels; manuscript locations refer to the current rendered revision.

Item	Reporting topic	Where reported	Compliance note
**1**	Title	Title, p. 1	Identifies the report as a scoping review.
**2**	Structured summary	Abstract, p. 1	Background, methods, results, and conclusions are reported.
**3**	Rationale	Introduction, pp. 1–2	Explains the distinction between device feasibility, diagnostic validity, clinical effectiveness, and care-pathway value.
**4**	Objectives	End of Introduction, p. 2	States four review questions covering distribution, translation stage, generalizability, and research priorities.
**5**	Protocol and registration	Methods 2.1, p. 2	Explicitly states that the protocol was not prospectively registered.
**6**	Eligibility criteria	Methods 2.1, p. 2	Reports eligible designs, settings, applications, and exclusions.
**7**	Information sources	Methods 2.2, p. 2	Distinguishes the auditable PubMed/MEDLINE flow through 31 July 2026 from reference screening and earlier Scopus, Web of Science, and Cochrane sensitivity histories.
**8**	Search strategy	Methods 2.2, p. 2; Supplement S1, “Search and audit”	Lists all audited PubMed terms and counts and explains why supplementary database histories were not pooled into the PRISMA flow.
**9**	Selection of evidence sources	Methods 2.3, pp. 2–3; Figure 1, p. 4	Reports independent screening, consensus resolution, record-level de-duplication, and selection flow.
**10**	Data charting process	Methods 2.3, pp. 2–3	Describes the combined-dataset re-audit and extracted fields.
**11**	Data items	Methods 2.3, p. 3; Supplement S1	Defines bibliographic identifiers, year, design, region, indication, sample, platform, outcomes, complications, evidence level, and limitations.
**12**	Critical appraisal of individual sources (optional)	Methods 2.4, p. 3	Reports design-matched appraisal using RoB 2, QUADAS-2, MINORS domains, or structured narrative criteria, without a non-comparable pooled score.
**13**	Synthesis methods	Methods 2.5, p. 3	Defines anatomical, study-architecture, and clinical-function axes and explains why meta-analysis was not planned.
**14**	Selection results	Results 3.1, pp. 3–4; Figure 1	Reports 2,650 identified records, 160 full texts, 56 exclusions, and 104 unique included primary publications.
**15**	Characteristics of evidence sources	Results 3.2–3.4, pp. 4–6; Figures 2–4; Table 1; Supplement S1	Reports anatomical, temporal, design, evidence-level, and platform characteristics.
**16**	Critical appraisal results (optional)	Results 3.3–3.7, pp. 5–7; Supplement S1, limitation column	Reports study architecture, evidence maturity, and study-level principal limitations without an artificial cross-design score.
**17**	Results of individual sources	Results 3.5–3.7, pp. 6–7; Supplement S1	Provides study-level findings in S1 and calibrated narrative results for diagnostic, therapeutic, workflow, and safety evidence.
**18**	Synthesis of results	Results 3.2–3.7, pp. 4–7; Figures 2–4; Table 1	Synthesizes evidence by joint, year, study category, platform, evidence level, application, safety, and maturity.
**19**	Summary of evidence	Discussion 4.1–4.4, pp. 7–9; Tables 2–3	Interprets the innovation-to-evidence gap, clinical positioning, generalizability, pathway value, and research priorities.

Item	Reporting topic	Where reported	Compliance note
**20**	Limitations	Discussion 4.5, p. 9	Reports database-audit, protocol, design heterogeneity, appraisal, reporting, publication, and industry limitations.
**21**	Conclusions	Conclusions, p. 9	Provides an evidence-calibrated readiness assessment and implications for practice and research.
**22**	Funding	Funding statement, p. 10	States that the review received no external funding.

Reference: Tricco AC, Lillie E, Zarin W, et al. PRISMA Extension for Scoping Reviews (PRISMA-ScR): Checklist and Explanation. Ann Intern Med. 2018;169:467–473. doi:10.7326/M18-0850.
